# Requirement for Cyclic AMP/Protein Kinase A-Dependent Canonical NFκB Signaling in the Adjuvant Action of Cholera Toxin and Its Non-toxic Derivative mmCT

**DOI:** 10.3389/fimmu.2019.00269

**Published:** 2019-02-19

**Authors:** Manuela Terrinoni, Jan Holmgren, Michael Lebens, Maximilian Larena

**Affiliations:** ^1^Department of Microbiology and Immunology, Institute of Biomedicine, University of Gothenburg Vaccine Research Institute (GUVAX), Sahlgrenska Academy at University of Gothenburg, Gothenburg, Sweden; ^2^Department of Organismal Biology, Uppsala University, Uppsala, Sweden

**Keywords:** NFκB pathway, adjuvant action, mucosal adjuvants, cholera toxin, mmCT

## Abstract

Cholera toxin (CT) is widely used as an effective adjuvant in experimental immunology for inducing mucosal immune responses; yet its mechanisms of adjuvant action remain incompletely defined. Here, we demonstrate that mice lacking NFκB, compared to wild-type (WT) mice, had a 90% reduction in their systemic and mucosal immune responses to oral immunization with a model protein antigen [Ovalbumin (OVA)] given together with CT. Further, NFκB^−/−^ mouse dendritic cells (DCs) stimulated *in vitro* with CT showed reduced expression of MHCII and co-stimulatory molecules, such as CD80 and CD86, as well as of IL-1β, and other pro-inflammatory cytokines compared to WT DCs. Using a human monocyte cell line THP1 with an NFκB activation reporter system, we show that CT induced NFκB signaling in human monocytes, and that inhibition of the cyclic AMP—protein kinase A (cAMP-PKA) pathway abrogated the activation and nuclear translocation of NFκB. In a human monocyte-CD4^+^ T cell co-culture system we further show that the strong Th17 response induced by CT treatment of monocytes was abolished by blocking the classical but not the alternative NFκB signaling pathway of monocytes. Our results indicate that activation of classical (canonical) NFκB pathway signaling in antigen-presenting cells (APCs) by CT is important for CT's adjuvant enhancement of Th17 responses. Similar findings were obtained using the almost completely detoxified mmCT mutant protein as adjuvant. Altogether, our results demonstrate that activation of the classical NFκB signal transduction pathway in APCs is important for the adjuvant action of both CT and mmCT.

## Introduction

Cholera toxin (CT) is a potent enterotoxin produced by *Vibrio cholerae* bacteria that, through its action on the intestinal epithelium in infected individuals, can cause the severe, often life-threatening diarrhea and fluid loss characteristic of cholera disease (1). CT is also a potent mucosal vaccine adjuvant that has been used extensively in experimental immunology (1, 2). However, in contrast to its enterotoxic activity which has been mechanistically well-defined, the signal transduction pathways through which CT exerts its strong adjuvant action remain incompletely understood. The lack of safe effective mucosal adjuvants is generally held as a main barrier for the development of a wider range of mucosal vaccines than the handful currently available, especially vaccines based on purified antigens (2). Understanding the molecular mechanisms of the adjuvant action of CT, which is generally held as the “gold standard” mucosal adjuvant, could clearly guide current efforts to develop alternative, non-toxic mucosal vaccine adjuvants for human use (3, 4).

Previous work by numerous groups has shown that CT promotes both cellular and humoral immune responses via its action mainly on antigen-presenting cells (APCs) in which it activates intracellular cyclic AMP—protein kinase A (cAMP-PKA)—and inflammasome-dependent pathways associated with expression, maturation, and release of IL-1β (5–13). This in turn indirectly, enhances both humoral and effector T cell responses (5, 13–16) and promotes Th17 as well as, Th2 and Th1 responses, the latter being more pronounced in mice than in humans. IL-1β is an important pro-inflammatory cytokine known to be induced via NFκB signaling by various well-established adjuvants, such as lipopolysaccharide (LPS), aluminum hydroxide, and saponins (17–19).

NFκB signaling is an important component of the immune system (20) involving multiple homodimeric or heterodimeric NFκB/Rel protein family members: p50/NFκB1, p52/NFκB2, p65/RelA, RelB, and c-Rel. The generation of an innate immune response via NFκB signaling occurs largely at the level of APCs, usually through the interaction between PAMPs (pathogen-associated molecular patterns) and membrane-bound or cytosolic PRRs (pattern recognition receptors) (21–24), leading to NFκB activation and translocation into the cell nucleus and subsequent NFκB-dependent increased expression of cytokines, chemokines and adhesion molecules important for APC activation and induction of the adaptive immune response. NFκB signal transduction mechanisms can be classified into the canonical (classical) or the alternative (non-classical) pathways. The canonical NFκB pathway is activated in cells in response to pro-inflammatory stimuli, such as LPS, TNF, or CD40L (25, 26), leading to activation of IKK (Inhibitor of Kappa B Kinase) complex, NFκB heterodimer p50-RelA (p65) release and nuclear translocation, DNA binding, and increased transcription of NFκB responsive elements. The alternative pathway, on the other hand, is activated by members of the TNF-receptor superfamily, such as the lymphotoxin receptor, B-cell activating factor, and CD40, and is dependent on the induction of NIK (NF-Kappa-B-Inducing Kinase) signaling, leading to release and nuclear translocation of mainly p52-RelB dimers (27).

The role, if any of NFκB signaling for the adjuvant action of CT is not well-understood. Earlier work reported that CT induces translocation of NFκB into the nucleus of both dendritic and intestinal epithelial cells, suggesting that NFκB signaling may be important in the adjuvant action of CT (28, 29). However, it remains to be determined whether the CT-induced nuclear translocation of NFκB in APCs will activate downstream functional pro-inflammatory NFκB signaling; whether this is mediated through a CT-induced activation of the cAMP-PKA pathway; and to which extent NFκB signaling is responsible for CT's adjuvant effect.

Here, we examine the role of NFκB in the adjuvant action of CT. Using studies of both murine and human APCs *in vitro* and immunization of NFκB^−/−^ as compared to wild-type mice *in vivo*, we demonstrate a strong, almost total dependence on NFκB signaling for CT's adjuvanticity. We further show that activation of NFκB by CT goes through the cAMP-PKA pathway; that the adjuvant effect is mediated via the classical, and not the alternative NFκB signaling pathway in APCs; and that CT-induced NFκB signaling is important in the expression of IL-1β, the key adjuvant cytokine for subsequent T cells activation. Since CT is too toxic for use as a vaccine adjuvant in humans, we also investigated the role of NFκB for the adjuvant activity on APCs of mmCT (multiple mutated CT), a recently developed non-toxic, yet adjuvant-active CT derivative generated by introducing multiple mutations in the toxic-active A subunit (30).

## Materials and Methods

### Adjuvants, Antigens, Polyclonal Stimulus, Protein Inhibitors

Purified cholera toxin (CT) was purchased from List Biological Laboratories, and mmCT, a non-toxic adjuvant-active derivative of CT, was prepared and purified in-house (30). The endotoxin contents determined by the Limulus assay were very low, 7.4 EU/mg protein for CT and 3.6 EU/mg protein for mmCT (13). Ovalbumin (OVA grade V; Sigma) was used as antigen for mice immunizations. Staphylococcal enterotoxin B (SEB; Sigma-Aldrich) was used as a superantigen polyclonal stimulus. Specific protein inhibitors used were H-89 (Sigma), a PKA inhibitor; caffeic acid phenethyl ester (CAPE, Sigma), a specific NFκB inhibitor; and aspirin, a COX-inhibitor.

### Mice

Female C57Bl/6 (B6) and NFκB p50^−/−^ mice [purchased from JAX Laboratories (31)], 6–8 weeks old when used for experiments, were housed under specific–pathogen–free conditions. All treatments and procedures were performed in accordance with the Swedish Animal Welfare Act (1988:534) and the Animal Welfare Ordinance (1988:539). The study was approved by the Ethical Committee for Laboratory Animals in Gothenburg, Sweden (Ethical permit number 56/13).

### Immunization and Collection of Specimens

Immunization of mice and collection and preparation of specimens for immunological assays were performed as previously described (32). Briefly, mice received two intragastric doses at an interval of 10 days of 1 mg OVA given alone or supplemented with 10 μg CT. Venous blood, small intestinal tissue and fecal pellets were collected 1 day before the first immunization and again at the time for sacrifice 10–12 days after the last immunization. Sera were prepared by removing cells from the blood samples by centrifugation, and stored at −20°C until analyzed. Fecal extracts were prepared by emulsifying five fecal pellets from each mouse in 500 μl of ice-cold PBS containing 0.1 mg/ml of soybean trypsin inhibitor (STI), 1% (w/v) bovine serum albumin (BSA, Sigma Aldrich), 25 mM ethylenediaminetetraacetic acid (EDTA), 0.035 mg/ml Pefabloc (Coatech AB) in PBS mixed 50–50% (v/v) with glycerol. Debris was removed by centrifugation (16,000 × g, 10 min, 4°C) and the supernatants were stored at −80°C until analyzed. Intestinal tissue was obtained by PERFEXT method (32). Briefly, the mice were perfused with 0.1% heparin–PBS solution immediately after sacrifice, followed by excision of ca 3-cm of the uppermost small intestine which was weighed before storage at −20°C in a PBS solution (1 ml per g of tissue) containing 2 mM phenylmethylsulfonyl fluoride, 0.1 mg/ml trypsin inhibitor from soybean (Sigma Chemical Co.), and 0.05 M EDTA. At the time for analysis, the samples were thawed, ice-cold saponin (Sigma) was added to a final concentration of 2% (wt/vol) to permeabilize cell membranes, and they were vortex-homogenized and kept at 4°C overnight. The tissue debris was spun down at 16,000 × g for 10 min, and the supernatant (referred to as intestinal tissue extract) was analyzed for antibody contentby ELISA.

### Cells and Cell Culture

#### Mouse DCs

Murine bone barrow-derived DCs (mBMDCs) were generated by culturing bone marrow (BM) cells for 9 days at 37°C in 5% CO_2_ in Iscove's Modified Dulbecco's Medium supplemented with 10% fetal calf serum, 1% l-glutamine, 1% gentamicin, 50 μM mercaptoethanol, and in the presence of 200 ng/ml Flt3-L (R&D systems, Biotechne).

#### Human APCs and T Cells

Peripheral blood mononuclear cells (PBMCs), CD14^+^ monocytes and CD4^+^ T cells were prepared from buffy coats of healthy human blood donors as previously described (13). DCs were purified from PBMCs using the “Blood Dendritic Cell Isolation Kit II” (Miltenyi Biotec), according to the manufacturer's protocol. Cells were maintained at 37°C with 5% CO_2_, in DMEM-F12 complete medium (Life Technologies) supplemented with 1% gentamicin (Sigma-Aldrich; 50 mg/ml) and 5% human AB^+^ serum (Sahlgrenska University Hospital blood bank).

#### Monocyte Cell Lines

THP1 cells and the THP1 ^Blue−NFκB^ monocyte cell line, carrying a stable integrated NFκB-inducible Secreted Embryonic Alkaline Phosphatase (SEAP) reporter construct used to analyze NFκB induction, were purchased from InvivoGen. The THP1 cells were maintained in supplemented RPMI medium (10% fetal bovine serum, 1% gentamycin, and 1% b-mercaptoethanol), and the THP1 ^Blue−NFκB^ cell line was maintained in the same medium supplemented with 100 μg/ml normicin (InvivoGen) and 100 U/ml-100 μg/ml pen-strep (InvivoGen). Cell handling and preparation were performed in accordance with the manufacturer's protocol (InvivoGen).

### Cell Treatments

#### Monocytes or Primary DCs—T Cells Co-culture

CD14^+^ monocytes (5 × 10^4^ in 200 μl/well) or total purified DCs (1 × 10^4^ in 200 μl/well) were stimulated with 1 μg/ml of CT or mmCT, or left untreated for 16 h in 96-well round bottom plates. When used in co-culture experiments with CD4^+^ T cells, the treated or untreated monocytes or DCs, after 3 washes with PBS, were then mixed with autologous CD4^+^ T cells (5 × 10^4^ monocytes or 1 × 10^4^ DCs and 1 × 10^5^ of autologous CD4^+^ T cells in 200 μl per well) together with SEB superantigen (10 ng/ml) and the cell mixture cultured for 3 days. Culture supernatants were then collected, and IL-17A cytokine levels were measured using an ELISA kit (Invitrogen). Control experiments using Polymyxin for inhibition of endotoxins demonstrated that the very low levels of endotoxin in CT and mmCT preparations used did not contribute to the cellular effects of these proteins (13).

For inhibition experiments, monocytes or DCs were treated with 20 μM H-89 or 20 μM CAPE added 1 h prior to the subsequent 16 h treatment with adjuvants.

For testing specific gene expression inhibition by small interfering RNAs (siRNAs), siRNAs with specificity for the RELA and RELB genes, respectively, and negative control ALL STAR siRNA were purchased from Qiagen. The siRNAs were diluted to a final concentration of 25 nM in culture medium without serum. HiPerFect Transfect reagent (Qiagen) was added according to the manufacturer's instructions and incubated for 10 min at 25°C for complex formation. The reagent mixture was then added to pre-seeded CD14^+^ cells, which were then transfected for 24 h at 37°C with 5% CO_2_. Cells were washed 3 times with PBS and then further incubated with 1 μg/ml CT or PBS for 16 h before further co-cultured with CD4^+^ T cells and analyzed for IL-17A production as described above.

*THP1*^*Blue*−*NF*κ*B*^ *cells*. THP1^Blue−NFκB^ cells (1 × 10^5^/well) were treated for 16 h with 1 μg/ml of CT or mmCT or 1 mM of the cAMP analog dcAMP or left untreated in cell culture medium in 96-well plates. Inhibition of PKA was tested by adding 20 μM H-89 1 h prior to the treatment with adjuvants. After incubation for 16 h, the cells were centrifuged at 350 × g for 5 min, and 20 μl of the cell supernatant was mixed with 180 μl pre-warmed SEAP detection reagent QUANTI-Blue (InvivoGen). After further incubation for 3 h at cell culture conditions, the levels of NFκB-induced SEAP were measured in a spectrophotometer at 620 nm.

### RNA Extraction, Sequencing, and Bioinformatics Analysis

Purified murine BMDCs (1 × 10^6^/ml) were left untreated or treated with 5 μg/ml of OVA given alone or with 1 μg/ml of CT for 2, 4, 16 h, washed three times with PBS, and stored at-70° C. Total RNA was extracted by RNeasy Mini-Kit (Qiagen), and was sent to Technology Center for Genomics & Bioinformatics, University of California, Los Angeles for cDNA library preparation (InteGenX Apollo 324 System) and sequencing using Illumina HiSeq 2000 sequencing system. Each sample generated a total of 80 to 100 million paired-end reads of 100 bp each.

TrimGalore!, version 0.3.5, was used to trim raw RNA-seq reads with the following criteria: quality cut-off of Q30, Illumina adapter trimming, and removal of reads that are < 30 bp and that are left unpaired. Reads were aligned with the reference genome using STAR software, and the aligned sequence reads were subsequently processed using SAMtools. In the end, a total of 75–105 million reads per sample was generated. To quantify gene expression, Htseq-count was used to tally the number of reads mapped to exonic regions of the genome. Transcript read counts that showed more than 2-fold difference between untreated and treated samples were then analyzed for function enrichment using Gene Ontology Biological Process category of DAVID Bioinformatics.

### ELISA Analysis

Serum and intestinal-mucosal antibody responses were determined by ELISA. High binding ELISA trays (Greiner) were coated overnight at 4°C with 1 μg/ml of OVA. Plates were washed 3 times and then blocked with 1% BSA for 1 h to minimize unspecific binding. Samples and a known sample used as a standard were included in each plates and titrated by 3-fold serial dilutions. Plates for IgG analysis were incubated for 90 min at room temperature and those for IgA determination for 4 h at 37°C. All plates were washed twice with 0.05% (v/v) Tween 20 in PBS and once with PBS. HRP-conjugated goat-anti-mouse IgG was added to the plates with serum samples and goat-anti-mouse IgA-HRP (Southern Biotech) to the plates with fecal, or small intestine extracts. The plates were incubated at 4°C overnight and after twice washing then developed with OPD for 20 min at which time the enzyme reaction was stopped with H_2_SO_4_ and absorbance values analyzed at 490 nm. Endpoint titers were determined as the extrapolated sample dilution giving an absorbance value of 0.4 above the no-sample background.

### Western Blot Analysis

THP1 monocytes cells (2 × 10^7^/5 ml) were left untreated or treated for 4 h with 1 μg/ml of CT at 37C with 5% CO_2_. Cells were harvested on ice and cytoplasmic and nuclear fractions were separated by using NE_PER Kit according to the manufacturer's instructions (NE_PER Thermo Scientific). The reagents were supplemented with protease inhibitors (Thermo Scientific). Total protein concentration was measured with a BCA Protein Assay Kit (Pierce). 10 μg of protein were denaturated in reducing sample buffer (NuPAGE LDS 4×; Novex®, Life Technologies) with addition of 2.5% β-mercaptoethanol (Sigma-Aldrich) and heated at 70° C for 10 min. Samples were separated by 4–12% Bis-Tris Gel SDS-PAGE (NuPage gels Novex®, Life Technologies) and then transferred onto a nitrocellulose transfer membrane (Millipore). After blocking with 5% non-fat milk in Tris-buffered saline (TBS) (150 mM NaCl, 3 mM EDTA, 50 mM Tris-HCl, pH 8.0) for 2 h, the membrane was thereafter immunoblotted using anti-p65 rabbit polyclonal antibody (Abcam), anti β-actin antibody (Cell Signaling—cytoplasmic housekeeping protein) and an anti-TBP antibody (Cell Signaling—nuclear housekeeping protein) at O/N 4°C. The membrane was then washed three times with TBST buffer (150 mM NaCl, 3 mM EDTA, 0.1% Tween-20, 50 mM Tris-HCl, pH 8.0) and incubated with horseradish peroxidase (HRP)-conjugated goat anti-rabbit antibody (Jackson ImmunoResearch) for 1 h at RT. After washing with TBST 2 times and with TBS 1 time, proteins were then visualized using the sensitive ECL Detection System (Pierce) according to the manufacturer's instructions.

### FACS Analysis

For flow cytometric analysis, mBMDCs (1 × 10^6^/ml) were left incubated with or without 1 μg/ml of CT or mmCT for 16 h. Cells were then washed and stained with the following murine antibodies: anti-CD11c BV711, anti-CD80 FITC, anti-CD86 APC (BD Biosciences), and anti-I-A/I-E Pacific Blue (BioLegend). After staining the cells were fixed in 4% paraformaldehyde and analyzed with an LSRII Flow Cytometer (BD Biosciences), and data were then analyzed with FlowJo software (Tree Star).

For intracellular staining of human IL-1β, PBMCs (2 × 10^6^/2 ml) were incubated with 1 μg/ml of CT or mmCT or medium only for 16 h, with or without prior addition of 20 μM CAPE, and the cells were then treated with brefeldin A (3 mg/ml; BD Biosciences) for another 4 h. Cells were washed, treated with AmCyan Live/Dead staining (Invitrogen), and then surface-stained with anti-CD4 A700, anti-CD3PerCP, and anti-CD14 FITC (BD Biosciences). After fixation and permeabilization with Cytofix/Cytoperm solution (BD Biosciences), cells were then finally stained with anti-IL-1β PE (BD Biosciences), washed and resuspended in FACS buffer prior to flow cytometric analysis.

### RT-PCR Assay

BMDCs (1 × 10^6^/ml) from B6 control mice and NFκB^−/−^ mice were left untreated or treated with 1 μg/ml of CT or mmCT for 16 h at 37°C in 5% CO_2_. Total RNA was extracted using the RNeasy Mini-Kit (Qiagen) and cDNA generated from 0.5 μg of total RNA using QuantiTect Reverse Transcription Kit (Qiagen). Customized quantitative real-time PCR was performed (SABiosciences) following the manufacturer's instructions. The data were normalized to Hypoxanthine Phosphoribosyltransferase 1 (HPRT) gene expression and analyzed using a web-based software package for the PCR array system (SABiosciences).

### Statistical Analysis

ANOVA or, when applicable, paired *t*-test were used for statistical comparisons; *p*-value of < 0.05 was considered statistically significant. In figures, *P*-values < 0.05, < 0.01, < 0.001, and < 0.0001 are represented by the symbols ^*^, ^**^, ^***^, and ^****^, respectively.

## Results

### NFκB Signaling Is Important for the *in vivo* Adjuvant Effect of CT in Mice

**Figure 1:**
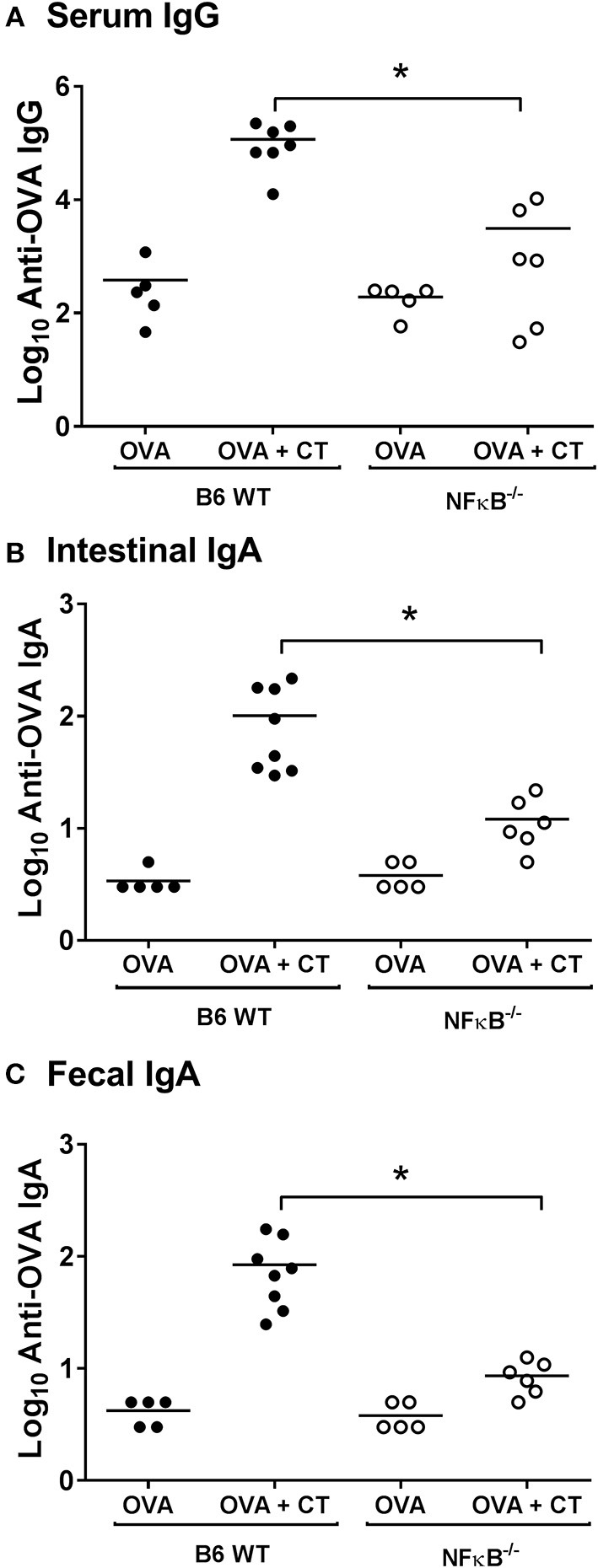
Absence of NFκB signaling impairs systemic and mucosal antibody responses after intragastric immunization with ovalbumin (OVA) with or without cholera toxin (CT). B6 [wild-type (WT)] or NFκB^−/−^ mice were immunized twice orally with 1 mg OVA together with or without 10 μg CT at an interval of 10 days. Levels of anti-OVA IgG in sera **(A)**, and anti-OVA IgA in intestinal tissue extracts **(B)**, or fecal extracts **(C)** 10 days after the last immunization were measured by ELISA. The data presented are pooled from two independent experiments showing similar results. ^*^represents *p* < 0.05 for indicated comparisons.

To examine the role of NFκB signaling on the adjuvant properties of CT *in vivo*, serum and intestinal-mucosal antibody responses were determined in NFκB^−/−^ and B6 WT control mice which were immunized with either OVA alone or OVA plus CT. As expected, there was a strong enhancement of both serum IgG and fecal and intestinal IgA anti-OVA responses in WT mice after immunization with OVA plus CT as compared to immunization with OVA alone (which latter in its turn increased anti-OVA serum IgG titers ca 10-fold above the pre-immunization background levels but did not significantly increase the fecal anti-OVA IgA levels, data not shown). In contrast, in the similarly immunized NFκB^−/−^ mice, the CT-induced enhancement was essentially lacking, being suppressed by ≥90% in comparison to the responses in WT mice (Figure 1). The results indicate that the adjuvant effect of CT on both mucosal and systemic humoral immune responses in mice is dependent on NFκB signaling.

### NFκB Signaling in Mouse DCs Is Upregulated by CT and Is Important for DC Activation and Stimulation of T Cells

The primary adjuvant action of CT appears to be to promote activation and antigen presenting capacity of DCs and other APCs (5, 33, 34). Transcriptomic analyses of BMDCs from WT mice exposed for different time periods to either OVA plus CT or for comparisons to OVA alone demonstrated that the transcripts for a large number of cytokines and other immunological activation markers were strongly upregulated by CT (Supplementary Figure S1). The levels of transcripts were usually higher after incubation for 16 h as compared to 2 or 4 h, but in some cases, most notably for IL-1β, IL-12, and CD83, the maximal gene expression occurred at the earlier time-points and had declined at 16 h. Among the genes that were upregulated in the CT-treated cells there was an especially strong increase in the IL-1β transcript level at 4 h, 22-fold for OVA + CT treated cells and >4-fold in OVA only treated cells; this agrees with previous studies demonstrating increased expression of this cytokine in CT-treated APCs and its important role for CT's adjuvant function (13, 33–36). In contrast, although the IL-12 transcript levels were slightly (< 2-fold) elevated at 2 and 4 h after OVA+CT treatment they did not differ from those after OVA only treatment and had essentially disappeared at 16 h, in accordance with previous reports that IL-12 expression is not specifically increased and may even be suppressed by CT (16, 37, 38).

Many of the CT-enhanced immune genes, e.g., IL-1α, IL-1β, CD80, and IL-6 are under NFκB regulation (39). Consistent with this and a previous report of CT-induced NFκB translocation to the nucleus of murine APCs *in vitro* (28), our transcriptomic analyses showed that treatment of murine DCs with CT promoted upregulation of gene sets associated with translocation of NFκB to the nucleus, effects that were prominent at both 4 h and at 16 h (Supplementary Figure S2).

**Figure 2:**
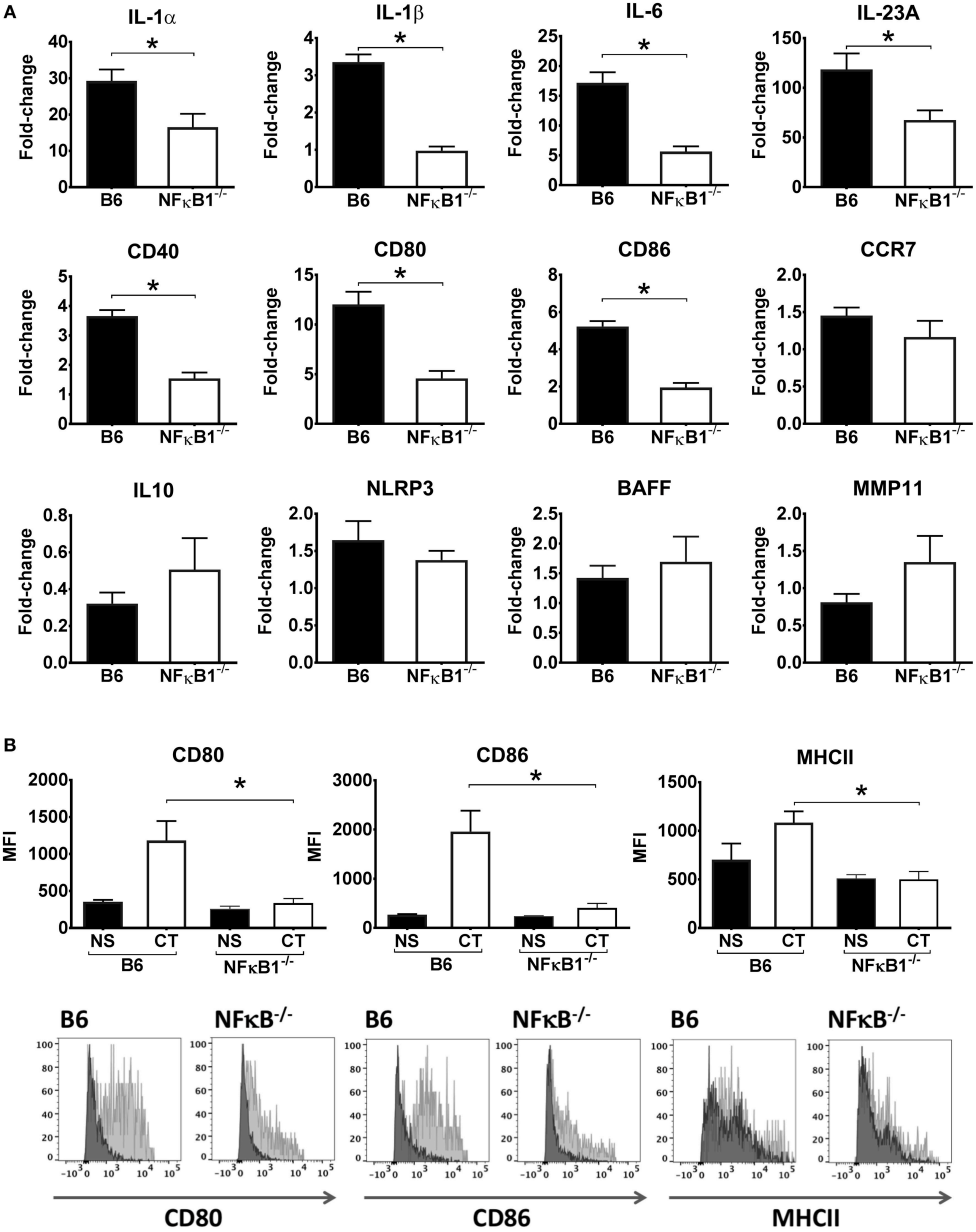
Lack of NFκB abrogates CT-induced increased gene expression for pro-inflammatory cytokines and other immune activation markers in DCs. BMDCs from B6 wild-type or NFκB^−/−^ mice were incubated in triplicates with 1 μg/ml CT for 16 h or left untreated. Purified total RNA preparations from the cells were used for inflammation focused gene expression studies by quantitative PCR. Bars represent means and SEMs of fold-change differences in gene expression between CT treated and untreated cells tested in triplicates **(A)**. Flow cytometric analyses **(B)** show median fluorescence intensity (MFI) and representative FACS histogram overlays of CD80, CD86, and MHCII expression in gated BMDCs from wild-type (B6) or NFκB^−/−^ mice incubated with either 1 μg/ml CT (light gray filled histogram) or only medium (NS), (dark gray filled histogram). ^*^p < 0.05 for comparisons between cells treated with CT and medium alone (NS) **(B)**.

To more directly examine the role of NFκB signaling in the activation of DCs by CT, CT-treated BMDCs from WT and NFκB^−/−^ mice were examined by RT-PCR to analyze gene expression for various cytokines and other immune-associated molecules. Consistent with our initial transcriptomic findings using WT DCs, the mRNA expression for IL-1α, IL-1β, IL-6, and IL-23 cytokines as well as for CD40, CD80 and CD86 surface co-stimulatory molecules were significantly increased in WT BMDCs treated with CT as compared to untreated, whereas they were enhanced to a much lower extent if at all in the NFκB^−/−^ BMDCs. Other examined genes, such as those for IL-10, BAFF, and MMP11 were not significantly increased by CT in either WT or NFκB^−/−^ BMDCs (Figure 2A). Further analyses by FACS supported that the CT-treated WT BMDCs had strongly increased expression of CD80 and CD86 as well as of MHCII on the cell surface, whilst the expression of these molecules on NFκB^−/−^ DCs was much lower and only modestly increased compared to the levels in untreated cells (Figure 2B). Thus, our data suggest that the CT-induced upregulation in BMDCs of many co-stimulatory molecules and pro-inflammatory cytokines associated with the adjuvant action of CT in mice is dependent on CT-induced activation of NFκB signaling.

### NFκB Signaling Is Also Required for the Adjuvant Action of CT on Human Immune Cells

Our attention next turned to examining the role of NFκB signaling in the adjuvant action of CT on human APCs. This was based on two main reasons. One was to learn whether our findings in mice would extend to humans, at least as testable on human APCs *in vitro*. Another reason was that while CT exhibits strong anti-proliferative effect on murine T cells which prohibits *in vitro* studies of CT-induced T cell activation in murine systems (40), this effect does not extend to human T cells, whose activation by CT-treated antigen-exposed human APCs can therefore easily be examined (13).

**Figure 3:**
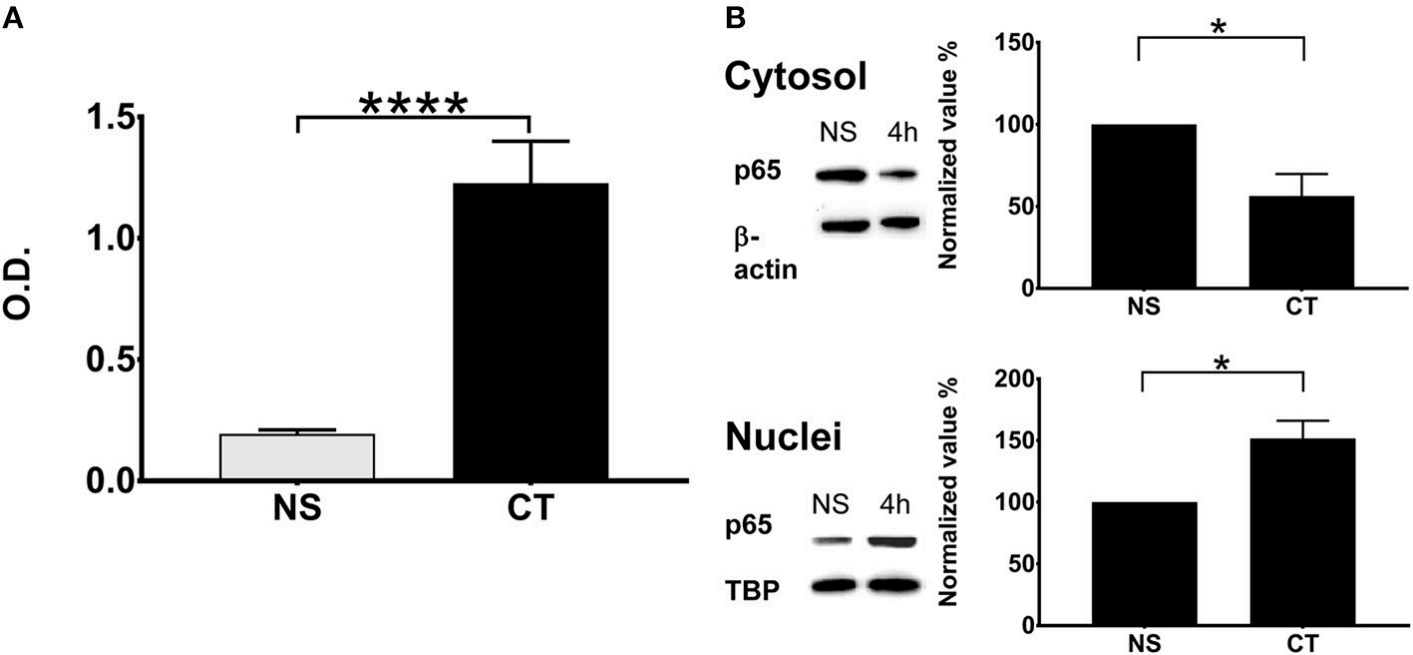
NFκB pathway in human monocytes is activated by CT with translocation of NFκB into the nucleus. **(A)** NFκB activation in the human monocyte cell line THP1^Blue−NFκB^ equipped with a NFκB reporter system is shown after treatment with 1 μg/ml CT for 18 h; O.D. is a measurement of a stable integrated NFκB inducible secreted embryonic alkaline phosphatase (SEAP) reporter construct that is directly proportional to the NFκB induction; bars show means plus SEMs of pooled data from three experiments of CT-treated and unstimulated (NS) cells tested in triplicates with ^****^ representing statistical significance at *p* < 0.00001. **(B)** Immunochemical evidence for CT-induced NFκB translocation into the nucleus. THP1 cells were incubated with or without 1 μg/ml CT for 4 h and proteins from cytosolic and nuclear fractions were separated on SDS-PAGE and subsequently immunoblotted with an anti-p65 antibody; β-actin immunoblotted with an anti-β-actin antibody served as a control housekeeping protein for the cytoplasm and immunoblotted TBP as a control housekeeping protein for the nucleus. Normalized values show the percentage ratios of p65 protein in relation to the housekeeping proteins after CT treatment as compared to a set value of 100% for unstimulated cells (NS). Bars show mean values plus SEMs for CT-treated and unstimulated (NS) cells tested in triplicates; ^*^ defines a statistically significant difference at *p* < 0.05.

We tested the effect of CT treatment on NFκB induction using a monocyte cell line (THP1^Blue−NFκB^) equipped with NFκB reporter system. Treatment of THP1^Blue−NFκB^ cells with CT resulted in very clear NFκB activation relative to untreated cells (Figure 3A). We also determined the translocation of canonical NFκB p65 from the cytosol to the nucleus in CT-treated THP1 cells. As shown in Figure 3B, cytoplasmic p65 was reduced at 4 h in CT-treated as compared to untreated cells whilst the nuclear amount of p65 protein was increased. This data demonstrates that CT treatment of human monocytes results in activation and nuclear translocation of NFκB canonical pathway.

**Figure 4:**
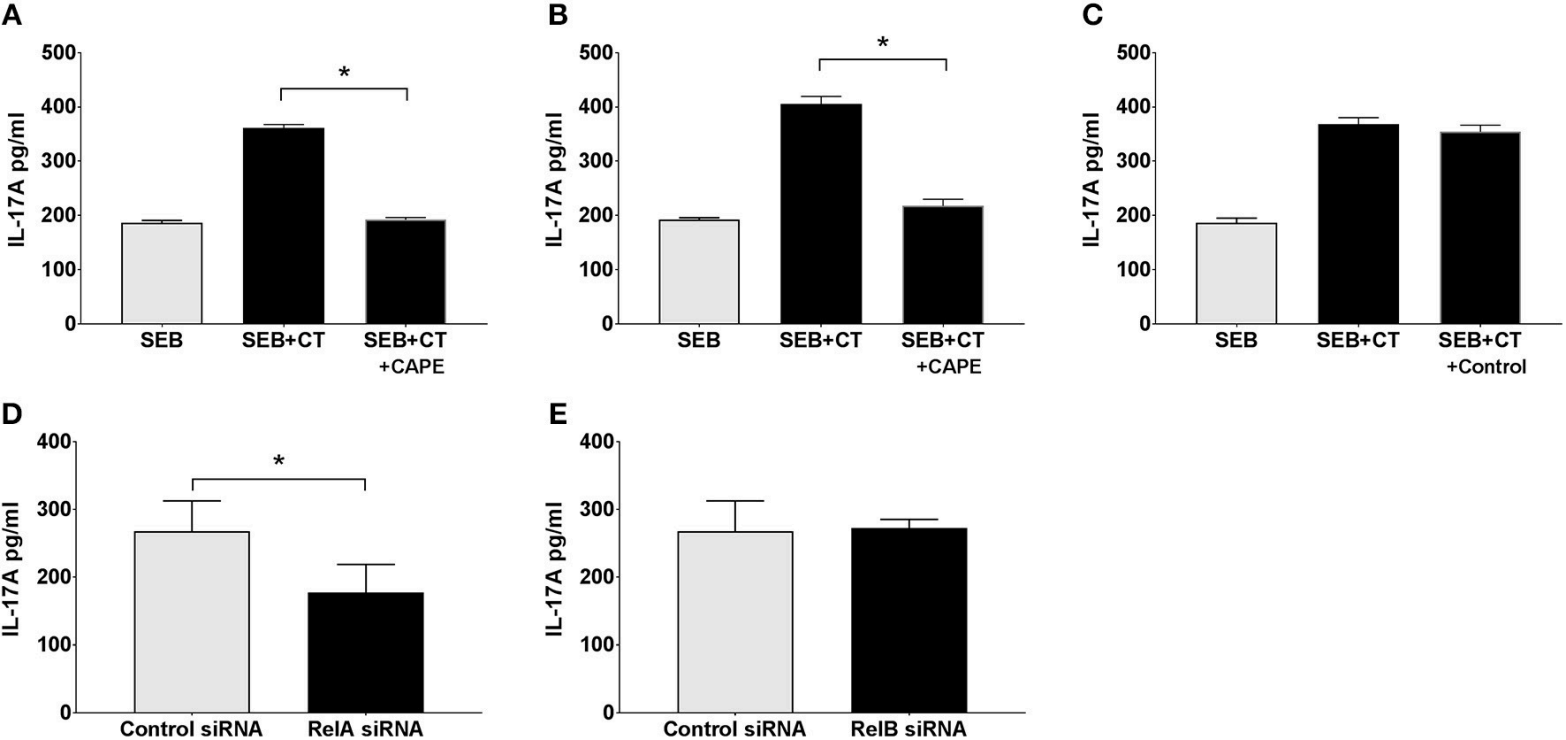
The canonical but not the alternative NFκB pathway in APCs is activated by CT and involved in its adjuvant activity. Purified human CD14^+^ monocytes **(A,C–E)** or DCs **(B)** were incubated for 1 h with the NFκB specific inhibitor CAPE **(A,B)**, or as a control with a COX inhibitor (Aspirin) **(C)**, or for 24 h with siRNAs specific for RelA involved in the canonical (classical) NFκB pathway **(D)**, All-Star Control **(D, E)**, or RelB involved in the alternative pathway **(E)**. Cells were then further treated for 16 h with 1 μg/ml CT or medium and then washed and co-cultured for 3 days with autologous CD4^+^ T cells plus SEB. Three separate experiments were performed, each including separate tests on cells from 3 to 5 individuals, and the data shown are the mean values plus SEMs of IL-17A levels in culture supernatants from all individuals measured by ELISA. ^*^represents *p* < 0.05 for compared values.

We examined whether NFκB signaling is required for the adjuvant action of CT on primary human APCs using a previously established co-culture system: purified human blood monocytes or DCs were incubated with CT or medium, and then after thorough washing the APCs were co-cultured with autologous CD4^+^ T cells in the presence of SEB superantigen, where after the levels of IL-17A, the predominant T cell cytokine increased by CT treatment of human APCs, were measured (13). In the present study, monocytes as well as DCs purified from human peripheral blood were either pre-treated with CAPE, a specific NFκB protein inhibitor, or left untreated, or as a further control treated were treated with Aspirin (a COX protein inhibitor) prior to the addition of CT or medium alone and the standard following procedures. The results show that while Th17 responses were significantly enhanced using CT-treated DCs or monocytes, they were significantly reduced when the CT-treated APCs had been pre-treated with the specific NFκB inhibitor (Figures 4A,B) but not with the control (COX) inhibitor (Figure 4C). The results support the importance of NFκB signaling for adjuvant effect of CT on human monocytes or DCs.

### The Adjuvanticity of CT Involves the Canonical, and Not the Alternative Pathway of NFκB Signaling

The NFκB signal induced by CT in THP1^Blue−NFκB^ demonstrates that CT stimulates classical/canonical NFκB signaling. However, NFκB signaling can also be mediated via alternative pathways (41). To examine whether either or both NFκB pathways are involved in the adjuvant action of CT, we undertook a modified monocyte-CD4^+^ T cell co-culture experiment. In this system, purified CD14^+^ monocytes were first transfected with silencing RNA (siRNA) specific for RelA involved in the canonical pathway or RelB involved in the alternative pathway, or with negative control siRNA (All-star siRNA) before being treated with CT. After washing, the monocytes were then co-cultured with purified CD4^+^ T cells together with SEB, and Th17 responses were measured. As expected, treatment of monocytes with the control siRNA did not interfere with the CT-induced enhancement of the IL-17A response (Figures 4D,E). Treatment with RelA-specific-siRNA (Figure 4D), but not with RelB-specific-siRNA (Figure 4E), on the other hand resulted in significant decrease of the CT-mediated IL-17A response. These findings suggest that activation of the canonical NFκB pathway is the main signal transduction mechanism involved in the adjuvant action of CT.

### CT-Induced NFκB Activation Is Mediated by cAMP-PKA Signaling

**Figure 5:**
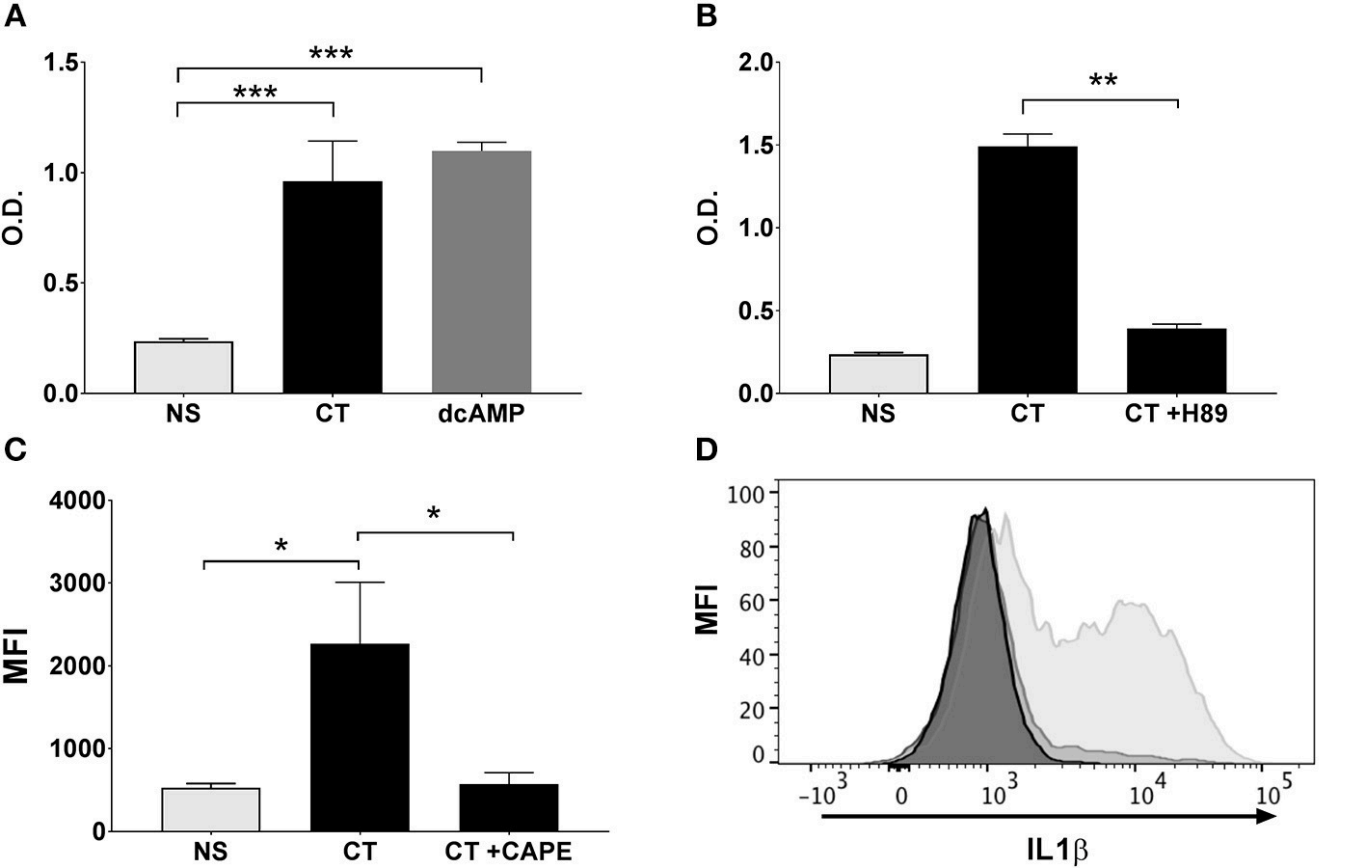
NFκB activation in APCs by CT is cAMP-PKA dependent and leads to the activation of IL-1 signaling. Human monocyte cell line (THP1^Blue−NFκB^) were treated in triplicates with the cAMP analog dcAMP **(A)** or the PKA inhibitor H-89 **(B)** prior to treatment with 1 μg/ml CT for 16 h. O.D. is a measurement of a stable integrated NFκB inducible secreted embryonic alkaline phosphatase (SEAP) reporter construct that is directly proportional to the NFκB induction. In **(C)**, PBMCs were treated in triplicates with or without CAPE for 1 h prior to a 16 h incubation with 1 μg/ml CT or medium only (NS), whereafter levels of intracellular IL-1β in CD14^+^ monocytes were analyzed by flow cytometry. Bars represent mean and SEM of median fluorescence intensity (MFI) for IL-1β. **(D)** shows representative ICCS histogram overlays of IL-1β expression in gated CD14^+^ monocytes treated with 1 μg/ml CT (light gray filled histogram), or with 1 μg/ml CT after preceding CAPE treatment (medium gray filled histogram), or with only medium (dark gray filled histogram). ^*^ represents *p* < 0.05 ^**^p < 0.01, and ^***^p < 0.001 for indicated comparisons. Data are from one of three independent experiments showing similar results.

Our previous work has demonstrated that the Th17-promoting adjuvant effect of CT on human cells *in vitro* involves cAMP-PKA signaling in monocytes and other APCs (13). Given the critical role of cAMP-PKA signaling and, as shown here, also NFκB signaling in the adjuvant action of CT, we investigated whether the activation of NFκB in CT-stimulated human monocytes is dependent on cAMP-PKA signaling. Treatment of THP1^Blue−NFκB^ cells with a cAMP analog (dcAMP) resulted in strong activation of NFκB signaling that is comparable in magnitude to that induced by CT (Figure 5A). Furthermore, treatment of the THP1^Blue−NFκB^ cells with a competitive inhibitor of cAMP-dependent PKA, H-89, prior to addition of CT abrogated the CT-induced NFκB activation (Figure 5B). These data support that NFκB activation by CT in human monocytes is dependent on PKA-cAMP signaling.

### CT-Induced Activation of NFκB in APCs Promotes IL-1 Signaling

IL-1 signaling by APCs has been found to be critical for the increase in Th17 responses by CT (36, 42–44). We have previously shown that inhibition of IL-1 signaling in human monocytes abrogated the Th17-promoting adjuvant effect of CT (13). To investigate whether the stimulation of IL-1 signaling in APCs by CT is dependent on NFκB, monocytes were treated with CT in presence or absence of the CAPE NFκB inhibitor, and intracellular IL-1β expression was then measured by flow cytometry. Consistent with our previous findings (13), CT induced strong upregulation of IL-1β in human monocytes, which was almost completely abrogated in cells pre-treated with CAPE (Figures 5C,D). These findings demonstrate that the CT-induced increase in IL-1β signaling in APCs is strongly NFκB-dependent.

### NFκB Signaling Is Also Required for the Adjuvant Activity of mmCT

**Figure 6:**
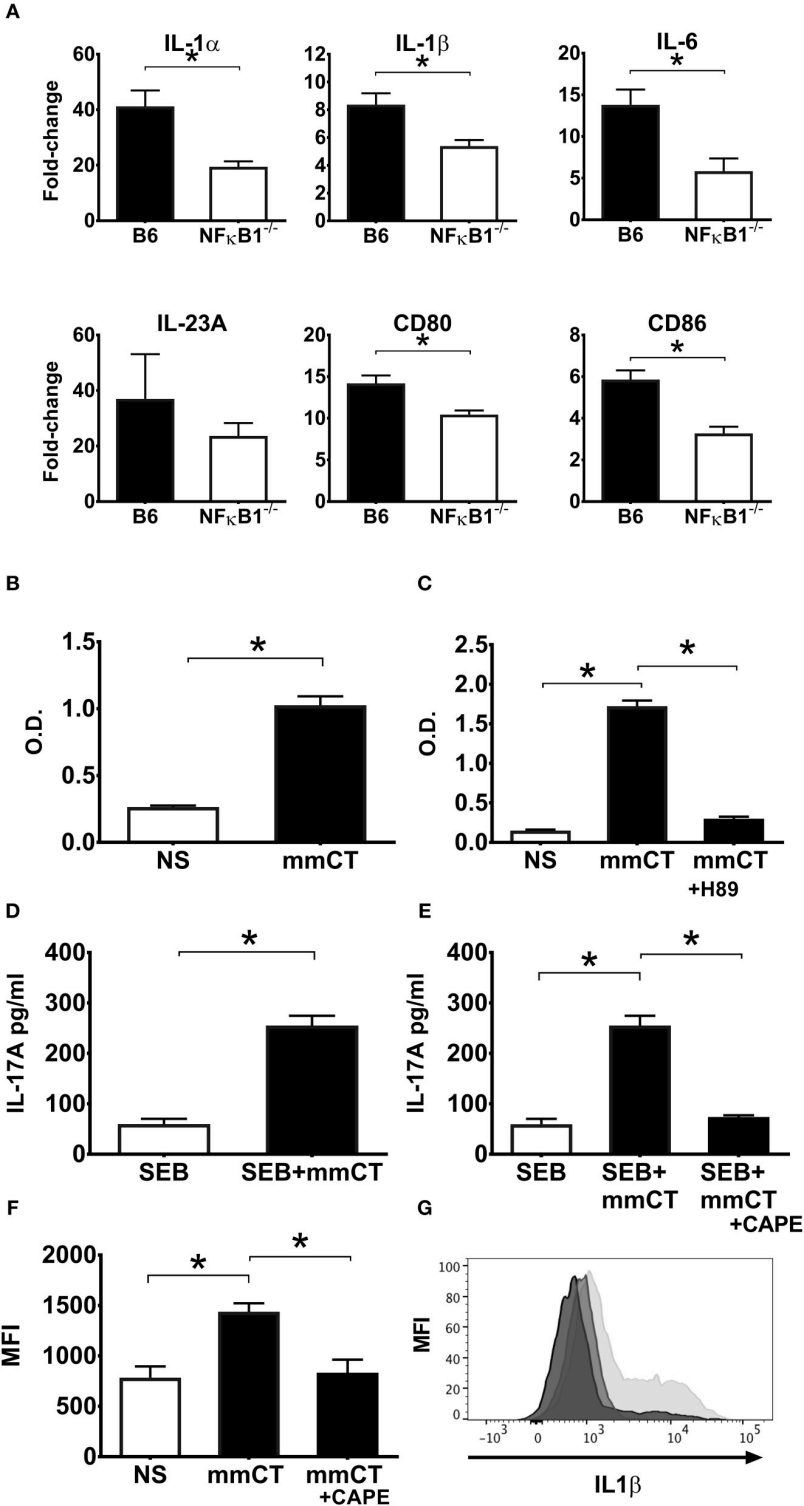
NFκB signaling is also required for the adjuvant activity of mmCT on mouse **(A)** and human APCs **(B–G)**. BMDCs from wild-type (B6) or NFκB^−/−^ mice were left untreated or were treated for 16 h with 1 μg/ml mmCT. Purified total RNA was prepared and used for subsequent determination of IL1α, IL1β, IL6, IL23, CD80, and CD86 gene expression by customized RT-PCR array from SABiosciences; bars represent means plus SEMs of fold-change differences in gene expression between mmCT treated and untreated samples **(A)**. Human monocyte cell line (THP1^Blue−NFκB^) were treated in triplicates with or without 1 μg/ml mmCT **(B)**, or in **(C)** with the PKA inhibitor H-89 for 1 h prior to treatment with 1 μg/ml mmCT for 16 h. In **(D)** purified CD14^+^ monocytes tested in triplicates were left untreated or were treated for 16 h with 1 μg/ml of mmCT or in **(E)** with the NFκB inhibitor CAPE for 1 h before the 16 h treatment with 1 μg/ml mmCT, whereafter the cells were, washed, and co-cultured for 3 days with autologous CD4^+^ T cells plus SEB, and secreted IL-17A in culture supernatants determined. Bars represent means plus SEM of IL-17A concentrations in culture supernatants measured by ELISA. In **(F)** PBMCs were treated in triplicates with or without CAPE for 1 h prior to 16 h treatment with 1 μg/ml mmCT, or medium (NS), and levels of intracellular IL-1β in CD14^+^ monocytes were then analyzed by flow cytometry. Bars represent means and SEMs of median fluorescence intensity (MFI) for IL-1β. **(G)** shows representative ICCS histogram overlays of IL-1β expression in gated CD14^+^ monocytes treated either with mmCT (light gray filled histogram), with mmCT after preceding CAPE treatment (medium gray filled histogram), or with only medium (dark gray filled histogram).^*^represents *p* < 0.05 for the indicated comparisons. Data are from one of three separate experiments showing similar results.

The toxicity of CT precludes its use as a vaccine adjuvant in humans, whereas the mutant molecule mmCT lacks detectable enterotoxicity and still has potent adjuvant activity (30). A series of experiment were performed to determine whether mmCT would display similar dependence on NFκB signaling for its adjuvant activity as demonstrated for CT in this study. First, gene expression analysis by RT-PCR on BMDCs from WT and NFκB^−/−^ mice treated with mmCT demonstrated a strong NFκB dependence for mmCT-induced transcription of both co-stimulatory molecules CD80 and CD86 and pro-inflammatory cytokines IL-1α, IL-1β, and IL-6 (Figure 6A). This was confirmed by flow cytometry analysis of mmCT-treated BMDCs that revealed reduced expression of CD80, CD86 as well as MHCII in NFκB^−/−^ BMDCs compared to the levels induced in WT BMDCs (data not shown).

The NFκB-dependence of mmCT's adjuvant activity was also demonstrated using human APCs. Treatment of THP1^Blue−NFκB^ cells with mmCT showed clear evidence of NFκB expression (Figure 6B). Further, pre-treatment of THP1^Blue−NFκB^ cells with the PKA inhibitor H-89 before the addition of mmCT resulted in abrogation of NFκB activation (Figure 6C), thus indicating a similar cAMP-PKA dependence of the mmCT-induced NFκB activation as seen with CT.

Moreover, co-culturing mmCT-treated monocytes with CD4^+^ T cells together with SEB showed that mmCT, similar to CT, induced a strongly enhanced Th17 response, which was abolished when the monocytes had been pre-treated with the NFκB inhibitor CAPE before the mmCT addition (Figures 6D,E). Likewise, intracellular IL-1β expression by human monocytes measured by flow cytometry, which was significantly increased by mmCT-treatment, was significantly reduced in mmCT-treated cells that had been pre-treated with CAPE, indicating that similar to CT, mmCT-induced IL-1β expression is dependent on NFκB signaling (Figures 6F,G).

Altogether, these data support and extend our previous work indicating that mmCT, despite its lack of detectable enterotoxicity and having 1,000-fold reduced ability to induce cAMP in target cells compared to CT, displays close similarity to CT with regard to its molecular mechanism of action. Both CT and mmCT induces NFκB signaling via a cAMP-PKA-dependent pathway, and the activation of NFκB leads to IL1β-dependent promotion of Th17 (and other cellular) responses.

## Discussion

This study identifies NFκB signaling as a key molecular pathway in the adjuvant action of both CT and the mutant CT derivative, mmCT. The latter molecule, despite its potent NFκB-inducing adjuvant activity, has no detectable enterotoxic activity, and should therefore, in contrast to CT, be possible to use as an adjuvant in humans. *In vivo* studies in WT and NFκB^−/−^ mice demonstrated that after oral immunization with a model protein (OVA) together with or without CT adjuvant, the lack of NFκB was associated with a >90% reduction in the capacity of CT to enhance OVA-specific mucosal IgA as well as systemic IgG responses. This was associated with a complete or marked reduction of the CT-induced increased gene expression for various immunostimulatory cytokines (IL-1α, IL-1β, IL-6, and IL-23) and co-stimulatory molecules (CD40, CD80, CD86) in NFκB^−/−^ BMDCs relative to WT. Since the p50 mutation in NFκB^−/−^ induces multifocal defects in the immune response (31) whereas CT is known to almost exclusively exert its adjuvant effect through activation of APCs, the pronounced reduction of immunostimulatory cytokines and co-stimulator molecules in NFκB^−/−^ DCs supports that the poor immune responses *in vivo* largely, if not exclusively reflect impaired APC activation by CT.

An important role for NFκB signaling in APCs for the adjuvant action of CT was also found when human immune cells were examined. In addition to demonstrating that the findings in mice extend to humans, at least as can be tested using human APCs *in vitro*, the consistent strong dependence on NFκB signaling for CT's adjuvant effects also on human APCs from multiple blood donors practically rules out that the effects observed to any significant degree were dependent on genetic or environmental factors, such as e.g., diet or microbiota.

In a human monocyte cell line THP1^Blue−NFκB^ with an inbuilt NFκB reporter system, CT increased NFκB expression as well as the translocation of NFκB into the nucleus. The functional significance of CT-induced NFκB signaling in human APCs for the adjuvant activity was indicated by a practically complete abrogation of CT's ability to promote SEB-induced T cell (Th17) responses when the NFκB signaling in the APCs, whether in monocytes or isolated DCs, was abolished by either a specific molecular inhibitor (CAPE) or siRNA. The requirement for NFκB signaling by CT is evidently restricted to canonical signaling, since siRNA inhibition of RelA but not of RelB prevented the enhancement of Th17 responses by CT. Interestingly, it was reported that the breakdown of OVA-induced oral tolerance in mice by oral co-administration of OVA with CT was associated with activation by CT of canonical NFκB pathway in Peyer's patches and mesenteric lymph nodes (45).

We investigated further the relationship between CT-induced cAMP/PKA signaling and NFκB signaling for the adjuvant effect of CT on APCs. Previous work has shown conflicting results reporting that cAMP/PKA signaling can either activate (46, 47) or inhibit (48, 49) NFκB, suggesting cell type- and/or context-dependent effects of cAMP/PKA signaling on NFκB activity. Our previous work has demonstrated that the predominant Th17-promoting adjuvant effect of CT on human immune cells *in vitro* is mediated via CT-induced cAMP-PKA signaling in monocytes and other APCs (13). Consistent with this, we demonstrate here that the induction of canonical NFκB signaling by CT appears to be mediated via cAMP/PKA activation. Using the THP1^Blue−NFκB^ cell line reporter system, we found strong NFκB activation when the cells were treated with a cAMP analog, whereas treatment of THP1^Blue−NFκB^ cells with a PKA inhibitor prior to addition of CT abolished the signal for NFκB activation. The detailed molecular mechanisms by which CT-induced cAMP/PKA signaling activates NFκB remains to be defined but may involve phosphorylation of RelA. PKA is known to phosphorylate Ser276 of RelA leading to nuclear translocation and increased transcriptional activity of NFκB. Besides Ser276, multiple other phosphorylation sites have been identified in RelA, which can serve as sites for direct or indirect interaction with cAMP-PKA signaling (47).

Importantly, the induction of NFκB signaling by CT in APCs triggers increased expression of IL-1β, an important pro-inflammatory cytokine for CT's adjuvant function (5, 35) and critical for the promotion of Th17 responses (13, 14, 36). This was clearly demonstrated when CT-stimulated monocytes were pre-treated with the NFκB inhibitor CAPE, in which case both the normal CT-induced increase in intracellular IL-1β and the promotion of Th17 responses in co-cultured CD4^+^ T cells were abolished.

A similar dependence on NFκB signaling for adjuvant activity as that shown for CT was also found for the practically non-toxic mmCT derivative. We have previously shown that the adjuvant function of mmCT on human APCs similar to CT is dependent on cAMP/PKA signaling (13) even though the cAMP levels induced by mmCT are 1,000-fold reduced compared to those induced by CT (30). We now extend this observation by demonstrating, both in murine and human APCs, that cAMP/PKA dependent NFκB signaling is important for the ability not only of CT but also of mmCT to increase expression of pro-inflammatory cytokines including IL-1β in APCs and, as tested in the human APC-T cell co-culture system, to functionally augment the development of Th17 cell response.

Similar to our previous findings on cytokine production in monocytes and IL-17 production from co-cultured T cells, the levels of NFKB activation and translocation by mmCT resembled those induced by CT, despite the much lower levels of cAMP that are induced by mmCT. Our previous conclusion that the low cAMP levels induced by mmCT are apparently “both sufficient and necessary” for its strong adjuvant effect clearly applies also to the activation of NFKB signaling in APCs by mmCT (13). This, however, does not exclude that there could still be differences between CT and mmCT in the way they may engage other as yet undefined pathways contributing to the adjuvant effect. In this regard, it is noteworthy that there are other enterotoxin derivatives, such as LTK63 and CTA1-DD whose adjuvant activity appears to be independent of cAMP (2, 50). When given intranasally to mice also the cholera toxin B subunit which does not induce any cAMP has significant adjuvant activity although less than for CT and mmCT (51, 52).

Altogether, as studied in both murine APCs *in vitro* and a mouse model *in vivo* as well as in human immune cells, our findings identify an important role of cAMP/PKA-dependent canonical NFκB signaling in APCs for the adjuvant activity of both CT and its practically non-toxic derivative mmCT.

## Data Availability

The RNA-seq datasets generated for this study can be found under the SRA BioProject ID: PRJNA517420.

## Ethics Statement

The study was approved by the Ethical Committee for Laboratory Animals in Gothenburg, Sweden (Ethical permit number 56/13).

## Author Contributions

MT, JH, MiL, and MaL conceived and designed the study. MT and MaL performed the experiments and analyzed the data. MT, JH, and MaL wrote the manuscript. All authors read and approved the final version of the manuscript.

### Conflict of Interest Statement

The authors declare that the research was conducted in the absence of any commercial or financial relationships that could be construed as a potential conflict of interest.
