# Supplementary material for: Requirement for Cyclic AMP/Protein Kinase A-Dependent Canonical NFκB Signaling in the Adjuvant Action of Cholera Toxin and Its Non-toxic Derivative mmCT
Source: Front Immunol. 2019 Feb 19;10:269. doi: 10.3389/fimmu.2019.00269 (PMC6389712; doi:10.3389/fimmu.2019.00269)
Supplement: Supplementary file 1 [file Data_Sheet_1.PDF]

Requirement for cyclic AMP/protein kinase A-dependent canonical NFκB signaling in the adjuvant action of cholera toxin and its non-toxic derivative mmCT

Manuela Terrinoni[1*], *Jan Holmgren*[1], *Michael Lebens*[1], *Maximilian Larena*[1, 2*]

Authors affiliations:

[1]Department of Microbiology and Immunology and University of Gothenburg Vaccine Research Institute (GUVAX), Institute of Biomedicine, Sahlgrenska Academy at University of Gothenburg, Box 435, SE-405 30 Gothenburg, Sweden.

[2]Department of Organismal Biology, Uppsala University, Norbyvägen 18C, SE-753 26, Uppsala, Sweden

*Corresponding authors: *Manuela Terrinoni*, Department of Microbiology and Immunology and University of Gothenburg Vaccine Research Institute (GUVAX), Institute of Biomedicine, Sahlgrenska Academy at University of Gothenburg, Medicinaregatan 7A, 41190, Gothenburg, Sweden. E-mail: manuela.terrinoni@gu.se Telephone: +46 317866226. *Maximilian Larena,* Department of Microbiology and Immunology and University of Gothenburg Vaccine Research Institute (GUVAX), Institute of Biomedicine, Sahlgrenska Academy at University of Gothenburg, Medicinaregatan 7A, 41190, Gothenburg, Sweden.. Email: Maximilian.larena@gu.se

Telephone: + 46 184712624

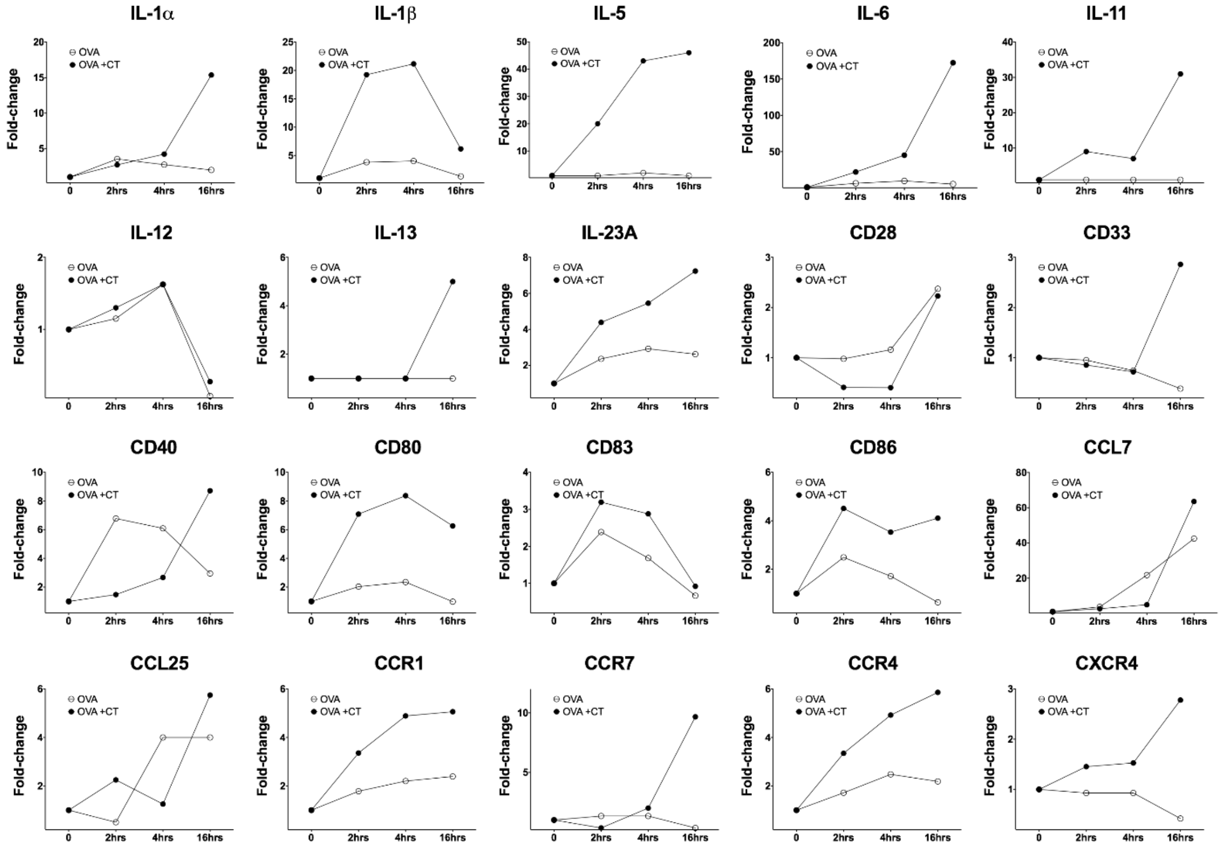

Figure S1. *CT strongly upregulates gene expression for immune associated cytokines, co-stimulatory molecules and other immunological markers in murine DCs*. Gene transcripts were measured in BMDCs that had been incubated with 5µg/ml OVA alone or with 1µg/ml CT for 2h, 4h, or 16h. Fold changes represent the transcript ratios between CT plus OVA treated and OVA only-treated DCs.

**CT 2hrs**		
Rank	*Pathway*	*Enrichment Score*
1	response to cAMP	1.64
2	sterol biosynthetic process	1.57
3	positive regulation of transcription	1.43
4	positive regulation of epithelial cell proliferation	1.18
5	Peptidyl tyrosine dephosphorylation	1.13

**CT 4hrs**		
Rank	*Pathway*	*Enrichment Score*
1	sterol biosynthetic process	2.59
2	tryptophan catabolic process	2.27
3	semaphorin-plexin signalling pathway	1.87
4	response to biotic stimulus	1.80
5	release of Cytochrome C from mitochondria	1.64
6	positive regulation of NFκB import into nucleus	1.60
7	cholesterol homeostasis	1.57
8	leukocyte homeostasis	1.15
9	peptidyl-tyrosine homeostasis	1.14
10	Protein tyrosine kinase activity	0.65

**CT 16hrs**		
Rank	*Pathway*	*Enrichment Score*
1	positive regulation of NFκB import into nucleus	1.65
2	positive regulation of IL6 production	1.59
3	circadian regulation and gene expression	1.16
4	neural tube development	1.11
5	metal ion transport	1.05
6	tumor necrosis factor-mediating signalling	0.92
7	Cell projection organization	0.90

Figure S2. The list of significantly up-regulated pathway–related genes at 2h, 4h and 16h of CT plus OVA-treated murine BMDCs were analyzed for functional enrichment using Gene Ontology Biological Process category of DAVID Bioinformatic Online Resource**.**
